# Ocular Surface Pathology in Patients Suffering from Mercury Intoxication

**DOI:** 10.3390/diagnostics11081326

**Published:** 2021-07-23

**Authors:** Pilar Cañadas, Yrbani Lantigua, Amalia Enríquez-de-Salamanca, Itziar Fernandez, Salvador Pastor-Idoate, Eva M. Sobas, Antonio Dueñas-Laita, José Luis Pérez-Castrillón, Jose C. Pastor Jimeno, Margarita Calonge

**Affiliations:** 1 IOBA (Institute of Applied Ophthalmobiology), University of Valladolid, 47011 Valladolid, Spain; yrbani.lantigua@hotmail.com (Y.L.); amalia@ioba.med.uva.es (A.E.-d.-S.); itziar.fernandez@ioba.med.uva.es (I.F.); spastori@ioba.med.uva.es (S.P.-I.); eva@ioba.med.uva.es (E.M.S.); pastor@ioba.med.uva.es (J.C.P.J.); calonge@ioba.med.uva.es (M.C.); 2 CIBER-BBN (Biomedical Research Networking Center Bioengineering, Biomaterials and Nanomedicine), Carlos III National Institute of Health, 28029 Madrid, Spain; 3 Department of Toxicology, Medical School, University of Valladolid, 47003 Valladolid, Spain; dueton@gmail.com; 4 Department of Internal Medicine, Medical School, University of Valladolid, 47003 Valladolid, Spain; uvacastrv@gmail.com

**Keywords:** cornea toxic effects, corneal esthesiometry, corneal innervation, in vivo confocal microscopy, mercury poisoning, occupational exposure, tear biomarkers, tear cytokines, corneal nerves, neurogenic dry eye

## Abstract

Purpose: To report the ocular surface pathology of patients suffering from acute/subacute mercury vapor intoxication. Design: Cross-sectional study. Participants: Male workers intoxicated with inorganic mercury referred for ophthalmic involvement and healthy control subjects. Methods: The following tests were performed: dry eye (DE)-related symptoms indicated by the ocular surface disease (OSDI) index questionnaire; tear osmolarity; analysis of 23 tear cytokine concentrations and principal component and hierarchical agglomerative cluster analyses; tear break-up time (T-BUT); corneal fluorescein and conjunctival lissamine green staining; tear production by Schirmer and tear lysozyme tests; mechanical and thermal corneal sensitivity (non-contact esthesiometry); and corneal nerve analysis and dendritic cell density by in vivo confocal microscopy (IVCM). Results: Twenty-two out of 29 evaluated patients entered the study. Most had DE-related symptoms (OSDI values > 12), that were severe in 63.6% of them. Tear osmolarity was elevated (>308 mOsms/L) in 83.4% of patients (mean 336.23 (28.71) mOsm/L). Corneal and conjunctival staining were unremarkable. T-BUT was low (<7 s) in 22.7% of patients. Schirmer test and tear lysozyme concentration were low in 13.6% and 27.3% of cases, respectively. Corneal esthesiometry showed patient mechanical (mean 147.81 (53.36) mL/min) and thermal thresholds to heat (+2.35 (+1.10) °C) and cold (−2.57 (−1.24) °C) to be significantly higher than controls. Corneal IVCM revealed lower values for nerve density (6.4 (2.94) n/mm^2^), nerve branching density (2 (2.50) n/mm^2^), and dendritic cell density (9.1 (8.84) n/mm^2^) in patients. Tear levels of IL-12p70, IL-6, RANTES, and VEGF were increased, whereas EGF and IP-10/CXCL10 were decreased compared to controls. Based on cytokine levels, two clusters of patients were identified. Compared to Cluster 1, Cluster 2 patients had significantly increased tear levels of 18 cytokines, decreased tear lysozyme, lower nerve branching density, fewer dendritic cells, and higher urine mercury levels. Conclusions: Patients suffering from systemic mercury intoxication showed symptoms and signs of ocular surface pathology, mainly by targeting the trigeminal nerve, as shown by alterations in corneal sensitivity and sub-basal nerve morphology.

## 1. Introduction

Mercury is a metallic element with a high potential for toxicity. Several public health disasters by mercury intoxication (also known as “mercury poisoning”) have been reported. The most well-known of these occurred in Japan in 1956 and was known as Minamata disease. Afterwards, the Minamata Convention on Mercury made the world aware of the environmental and public health issues that mercury pollution represents [1]. The most frequent human exposure to water-soluble forms of mercury, such as mercuric chloride or methylmercury, is caused by ingestion of any form of mercury, e.g., contaminated fish, or by inhalation of mercury vapor as an occupational exposure, including coal burning and mining, especially of mercury and gold [1].

Mercury can potentially impair the function of any organ or any subcellular structure because all forms of it have the potential to poison cellular function by altering the tertiary and quaternary structure of proteins and by binding with sulfhydryl and selenohydryl groups. Thus, exposure to different forms of mercury has been associated with adverse health effects [2]. Depending on the form of mercury (organic or inorganic), toxicity varies with the dose and the rate of exposure. Intoxication due to inhalation of mercury vapor, a form of inorganic mercury, produces the most harmful effects, as up to 80% of the inhaled mercury is absorbed and rapidly oxidized to other forms. Oxidized mercury vapor becomes lipid soluble, so the potential exists for bioaccumulation in the renal cortex, liver, and especially in the brain, where it has been estimated that the half-life of mercury can be as long as 20 years [3]. Even though the principal target organ of mercury vapor is the brain, functional degradation of peripheral nerves, and of the renal, immune, endocrine, and muscle systems, and several types of dermatitis, have been described [4].

Numerous ophthalmic findings due to mercury toxicity and its action on the retina and optic nerve have been reported for both chronic and acute exposures. These include decreased night vision, decreased color vision and contrast sensitivity, central visual impairment, progressive visual field constriction, and optic atrophy [5]. Less frequent symptoms and signs such as photophobia, blepharospasm, nystagmus, and mercury deposits on the anterior capsule of the lens (mercurialentis) and corneal stroma have also been reported in some cases of chronic intoxication [5].

In the 1970s, ocular surface alterations such as conjunctivitis and unspecific ocular irritation produced by chronic [6] and acute [7] exposure to methylmercury were reported in experimental animals. One previous study demonstrated that metallic mercury in the conjunctiva of rabbits increased the number of lymphocytes and macrophages and was also associated with increased amounts of altered mucus [8]. However, there are no published studies reporting human ocular surface effects.

At the end of 2012, 49 workers in Northern Spain were accidentally exposed to dangerous levels of mercury vapor. Blood and urine levels of mercury were above the recommended biological limits for occupational exposure, and acute and subacute (acute/subacute) mercury vapor intoxication was confirmed. Most of these patients were referred to the University of Valladolid, Spain, and those with ophthalmic complaints were referred to the Institute of Applied Ophthalmobiology (IOBA), University of Valladolid, Valladolid, Spain.

In this study, we describe in detail the ocular surface alterations found in the patients with acute/subacute mercury vapor intoxication, including mechanical and temperature sensitivity of the cornea, confocal microscopy findings, and the tear levels of several cytokines.

## 2. Methods

### 2.1. Patients

This cross-sectional study was conducted at the Institute of Applied Ophthalmobiology (IOBA) of the University of Valladolid, Spain, and approved by the Institutional Review Board and by the Ethics Committee of the Valladolid University Clinical Hospital. All enrolled patients were informed of the aims of the study, and their written consent was obtained according to the guidelines of the Declaration of Helsinki.

Acute/subacute mercury vapor intoxication occurred in 49 male workers accidentally exposed to dangerous mercury vapor levels for 14 consecutive days in a metal manufacturing plant in Northern Spain between 19 November and 2 December 2012. In the days after exposure, many of the workers reported physical complaints, mainly asthenia, headache, epigastric and abdominal pain, cough, bitter taste, dental pain, and gum inflammation and bleeding. Some of the workers also had ocular symptoms such as irritation, redness, burning, foreign body sensation, and light photophobia. Blood and urine mercury levels were measured during the second week of the exposure, and were above the recommended biological limits for occupational exposure, reaching maximum range levels between 252.62 and 507.47 μg/L in blood (normal, <10 μg/L) and between 93.61 and 245.57 μg/g creatinine in urine (normal, <30 μg/g creatinine). Some weeks before occupational exposure, random urine samples (*n* = 17) detected mercury levels below 3 μg/g creatinine in those workers. The diagnosis of acute/subacute intoxication with mercury vapor was confirmed by the post-exposure clinical analyses.

Between September 2013 and December 2014, 44 of the original 49 intoxicated patients were referred to the Clinical Toxicology Referral Unit of the Institute of Medical Sciences (ICIME) at the University of Valladolid for an independent evaluation. Of those, 29 patients had ocular symptomatology and were immediately referred to IOBA and evaluated for enrollment. All referred patients had vision and retinal-related symptoms, which are being reported by our colleagues (manuscript submitted for publication), in addition to ocular surface-related symptoms. 

All of the referred patients received medical care and were evaluated for inclusion in this study. The only inclusion criterion was that patients had to have reported their ocular surface symptoms after the mercury intoxication. The exclusion criteria were (1) pre-existing ocular surface symptoms, as reported by each patient; (2) use of artificial tears or any other topical medication before mercury intoxication; (3) use of any topical medication other than artificial tears 4 weeks before enrollment or 3 months before enrollment in the case of either topical cyclosporine or tacrolimus; (4) use of contact lenses in the previous 3 months; (5) any other ocular surface disease; or (6) any previous ocular surgery. The healthy controls used were 22 males from our files who had a non-significant age difference with respect to patients (42.0 ± 7.6, range, 28–56 years). These normal controls had no other ocular or systemic disease, were non-contact lens wearers, used no ocular medications or artificial lubrication, were asymptomatic, and their ocular surface evaluation (slit-lamp examination, tear stability, ocular surface integrity, and tear production) was within normal limits (see normal limits below).

### 2.2. Clinical Evaluation and Tear Sample Collection

Patients were evaluated at the Ocular Surface and Immunology Unit of IOBA. Both eyes were evaluated in all patients between 10 a.m. and 2 p.m. The temperature of the examination room was always set at 19–21 °C, and the relative humidity was between 50–60%. Clinical evaluation and subjective tests were always performed by the same examiner, in a single visit, with a 2–5 min interval between tests that were performed in the sequence detailed below.

(1) Ocular surface-related symptom questionnaire. The Ocular Surface Disease Index (OSDI), consisted of 12 questions that evaluated symptoms experienced in the preceding week. The questionnaire was self-administered and scored on a range of 0 to 100. Based on their OSDI score, each patient was categorized as having no symptoms (score 0–12) or as having mild (score 13–22), moderate (score 23–32 points), or severe (score 33–100) ocular surface-related symptoms [9].
  After completing the OSDI questionnaire, we asked each patient which eye he considered the most symptomatic. That eye was used for tear sampling, osmolarity measurement, esthesiometry, microscopy, and in statistical analyses of the clinical tests. If both eyes were equally symptomatic, the eye was selected by computer-generated randomization.
(2) Tear sample collection for molecular analysis. We followed our previous protocol in which unstimulated basal tear samples were collected non-traumatically from the external canthus, avoiding reflex tearing as much as possible [10]. One microliter of tear sample was collected with a glass capillary micropipette (Drummond, Broomall, PA, USA). Each sample was then diluted 1:10 in a sterile collection tube containing ice-cold Cytokine Assay Buffer (Milliplex, Millipore Merck Life Science SLU, Madrid, Spain). Tubes with tear samples were kept cold (4 °C) during collection and then stored at −80 °C until assayed.
(3) Tear osmolarity. The osmolarity of each tear sample was assessed by the TearLab osmometer (TearLab Corporation, San Diego, CA, USA) analysis of a 50-nL tear sample collected from the external canthus. Although the cutoff value for abnormal tear osmolarity can vary according to different authors, following the manufacturer’s indications, values above 308 mOsm/L were considered higher than normal [11].
(4) Conjunctival bulbar hyperemia. The nasal and temporal conjunctivas were assessed independently with a slit-lamp biomicroscope (SL-8Z; Topcon Corp, Tokyo, Japan) based on the Efron scale (0–4 score). The final score was the average of the nasal and temporal values [11].
(5) Tear break-up time (T-BUT). Tear stability was assessed by T-BUT. After instillation of 5 μL of 2% sodium fluorescein into the inferior fornix, the time between the last of three blinks and the appearance of the first dry spot was measured three times, and the mean value was recorded. Values of less than 7 s are currently considered abnormal [11].
(6) Ocular surface integrity. Corneal and conjunctival integrity were evaluated with fluorescein and lissamine green staining, respectively. The Oxford scheme (0–5 score) for grading the staining of both areas was used [11]. Corneal fluorescein staining was evaluated 2 min after instillation of 5 μL of 2% sodium fluorescein. The cobalt blue filter of the slit lamp was used with a yellow Wratten no. 12 filter (Eastman Kodak, Rochester, NY, USA) over the light source. Nasal and temporal bulbar conjunctival staining was evaluated using lissamine green strips (GreenGlo; HUB Pharmaceuticals, LLC, Rancho Cucamonga, CA, USA) wetted with 25 μL sodium chloride and then gently applied into the inferior fornix.
(7) Tear production. Tear production was assessed with two different tests: tear lysozyme level assay and Schirmer’s test without topical anesthesia. The tear lysozyme concentration test is routinely performed in our institution as a marker of aqueous-deficient dry eye (DE), as previously detailed [12]. Briefly, tears were sampled by applying a 5-mm diameter filter paper disc in the inferior fornix, and the eye was held closed for 1 min. The assay was carried out with the *Micrococcus lysodeikticus* (ATCC 4698, M3770; Sigma-Aldrich, St. Louis, MO, USA) agar diffusion assay in Mueller Hinton agar plates (Bio Merieux, Marcy l’Etoile, France). Lysozyme concentration was calculated from a standard curve of the inhibition hallux generated with several concentrations of commercial lysozyme (ATCC 4698, L6876; Sigma-Aldrich). Values of less than 1000 μg/mL were considered abnormal and thus indicative of low tear production [12].
  Immediately after the lysozyme tear production assay, the Schirmer test was performed by placing a sterile strip (I-DEW tear strips, Entod Research Cell UK, Ltd., London, UK) in the lateral canthus of the inferior lid margin. Subjects were asked to maintain eye closure during the test, and the length of wetting was measured after 5 min. Results below 5-mm length were considered abnormal [11].
(8) Corneal sensitivity. Corneal sensitivity was measured with a prototype Belmonte’s non-contact gas esthesiometer as previously reported by our group [13]. The corneal threshold for mechanical and thermal (cold and heat) sensitivities was determined in the central cornea using the method of levels. Three-second air pulses of adjustable flow rate and temperature were applied to the center of the cornea for determining corneal sensitivity thresholds. The mechanical threshold was always determined first. The probe of the esthesiometer was mounted on a base adapted to a slit lamp. Subjects were instructed to look at a fixation target at 3 m, and the tip was placed perpendicular to the corneal apex, 5 mm from the surface, measured with a transparent ruler. Mechanical stimulation consisted of a series of variable flows of medicinal air (0–200 mL/min). Air was heated at the tip of the probe at 50 °C so that it reached the ocular surface at 34 °C to prevent a change in corneal temperature caused by the airflow. Thermal thresholds were determined by heating or cooling the air to produce changes in basal corneal temperature of ± 0.1 °C, with a 10 mL/min flow below the mechanical threshold. A noise (a click produced by opening the gas valve) indicated the start of the pulse. Immediately after each stimulation pulse, the subject was asked to report the presence or absence of sensation. The order of heat and cold threshold measurement was randomized. Results were compared with a control group of 22 healthy males whose characteristics have been described above.
(9) In vivo confocal microscopy (IVCM). Laser scanning IVCM of the cornea was performed using the Rostock cornea module of the Heidelberg Retina Tomograph 3 (Heidelberg Engineering GmbH, Heidelberg, Germany). Before examination, a drop of anesthetic was instilled, and an eye speculum was used to keep the lids wide open. A drop of Viscotears Gel (Carbomer 980, 0.2%; Novartis Farmacéutica S.A., Barcelona, Spain) was deposited on the objective lens, thus avoiding direct contact of the TomoCap with the cornea. At least three good quality, non-overlapping images from the sub-basal nerve plexus of the central cornea were obtained using sequence and/or volume scans, and were used for the analysis. Each image was comprised of 384 × 384 pixels covering an area of 400 × 400 μM (0.16 mm^2^) with a transverse optical resolution of 2 μM, an axial optical resolution of 4 μM, and an acquisition time of 0.024 s.
  For IVCM image analysis, two masked observers analyzed the following in the three images: (1) nerve morphology parameters of density, length, branching density, and grade of tortuosity; (2) density of dendritic cells; (3) presence of neuromas; and (4) reflectivity from the confocal images, as an index of optic densitometry or transparency of cornea [14]. The mean value between the two observers for each parameter was computed for statistical analysis.
  Nerve density (n/mm^2^) and length (mm/mm^2^) were measured using the plugin NeuronJ (http://www.imagescience.org/meijering/software/neuronj/ accessed on 25 May 2021) from the ImageJ and provides quantification. The number of nerve branch points and dendritic cells (identified in the sub-basal nerve plexus by their distinctive features, i.e., bright cell bodies with dendritic form structures), were manually determined using the multipoint tool of the ImageJ software, and the densities calculated (n/mm^2^) as described in a previous study [15]. The grade of nerve tortuosity was evaluated according to the scale (0–4) reported by Oliveira-Soto and Efron [16] for main nerves. The histogram of each image based on the ImageJ plugin was used to obtain the mean reflectivity or optic densitometry [14]. These parameters were compared with well-established values for normal corneas and performed with the same type of confocal microscope. Specifically, we used data from Giannacare et al. [17] for nerve length, and from our group for nerve density, density of nerve branches, density of dendritic cells, [15] and nerve tortuosity and reflectivity [14].
(10) Analysis of tear cytokine concentrations. A commercial customized immunobead-based array was used to analyze the concentration of 23 cytokines and chemokines in tear samples with a Luminex IS-100 (Luminex Corporation, Austin, TX, USA). The concentrations of interleukin (IL)-1β IL-1 receptor antagonist (IL-1RA), IL-2, IL-4, IL-5, IL-6, chemokine (C-XC motif) ligand 8 (CXCL8)/IL-8, IL-9, IL-10, IL-12p70, IL-13, IL-17A, chemokine (C-X-C motif) ligand 10 (CXCL10)/interferon gamma-induced protein 10 (IP-10), chemokine (C-C motif) ligand 2 (CCL2)/MCP-1, chemokine (C-C motif) ligand 3 (CCL3)/MIP1-αchemokine (C-C motif) ligand 5 (CCL5)/regulated on activation, normal T-cell expressed and secreted (RANTES), chemokine (C-C motif) ligand 11 (CCL11/eotaxin-1), chemokine (C-X3-C motif) ligand 1 (CX3CL1)/fractalkine, interferon gamma (IFN)-γ, matrix metalloproteinase-9 (MMP-9), tumor necrosis factor (TNF)-α, epidermal growth factor (EGF), and vascular endothelial growth factor (VEGF) were measured simultaneously with a customized 23-plex SPR assay (SPR591 HCYTO- 60K, 23X-Milliplex). The samples were analyzed following the manufacturer’s low volume sample protocol that only uses 10 μL of sample/standards per assay, as previously described [10]. Data were stored and analyzed with the “Bead View Software” (Upstate-Millipore Corporation, Watford, UK). Standard curves were used to convert fluorescence units to molecule concentrations (pg/mL). The minimum detectable concentration, based on manufacturer specifications, was 1.2 pg/mL. Molecules that were detected in less than 30% of the samples were not statistically analyzed any further. Results were compared with a control group of 22 healthy males from our files, whose characteristics have been described above.

### 2.3. Statistical Analysis

Statistical analysis was performed using R software version 3.4.1 (R Foundation for Statistical Computing, Vienna, Austria). Significance level was set at 5%. Quantitative data were summarized as means and standard deviations (SD). Ordinal values were described using medians and interquartile ranges [IQR], unless otherwise specified in the text. The normality assumption was checked by the Shapiro–Wilk test.

Data from the study group were compared to the control group. Student’s t-tests for two independent samples were used to compare differences between mean values. Levene’s test was used to check homogeneity of variance, and Welch’s test was used when this assumption was not valid. When normality assumptions were not supported, the nonparametric alternative, Mann–Whitney U test, was performed.

For tear cytokine/chemokine analysis values out of range, the values were imputed by the regression on order statistics method. This technique performs a regression to impute low values assuming a log-normal distribution. The detection rates in the study and control groups were compared using equality of two proportions test. Cytokine expression data were transformed using the logarithmic base 2 scale. Expression levels in the study group were compared with levels in a control group from our database. In addition, principal component and hierarchical agglomerative cluster analyses were used to explore correlation patterns among cytokine levels in the study group. To facilitate the interpretation of the clustering result, a profile analysis was conducted, testing the differences among clusters by the same methodology as the one used to compare the study and control groups.

## 3. Results

All of the 44 patients examined at the University Clinical Toxicology Unit had erethism mercurialis and peripheral nervous system alterations that were confirmed by electrophysiology. Twenty-nine of the patients (65.9%) had visual complaints and were consequently referred to IOBA. The retinal and neurophthalmic pathological findings for these patients are under study by our colleagues. The ocular surface pathology of these patients has not been published and is the subject of this report.

Of the 29 patients with visual complaints, one was evaluated but excluded due to previous corneal refractive surgery in both eyes, and six others were clinically evaluated but declined to participate in this study. Therefore, a total of 22 male patients were finally included in this study and had complete ocular surface assessments. The mean age of the study group was 42.0 (7.6) (range, 28–56) years.

### 3.1. Clinical Tests

Results from the clinical tests are shown in Table 1. Mean OSDI values were abnormal (>12) in all patients except in patient 18, who had a score of 12. Based on this questionnaire, the majority of patients, 14 (63.6%), had severe DE-related symptoms, 4 (18.18%) had moderate symptoms, and 3 (16.6%) had mild symptomatology. Conjunctival hyperemia and ocular surface integrity (corneal fluorescein and conjunctival lissamine green staining respectively) findings were unremarkable. Tear film stability, evaluated with T-BUT, was under normal values (7 s) in five (22.7%) patients. Tear production was low in three (13.6%) patients based on Schirmer test and in six (27.3%) patients based on tear lysozyme concentration. However, tear osmolarity was abnormally elevated (>308 mOsm/L) in 83.4% of the patients. 

### 3.2. Corneal Sensitivity

Mechanical threshold and thermal thresholds for heat and cold were assessed in 21 of the 22 patients (Table 1 and Table 2) and in 22 control subjects (Table 2). Patient 11 refused to have corneal esthesiometry performed. All of the sensitivity thresholds were significantly higher in the mercury-intoxicated patients, indicating that their corneal sensitivity was decreased.

### 3.3. IVCM Findings

IVCM was performed in 15 out of the 22 patients. This evaluation was not possible for technical reasons in four patients and three others did not cooperate enough to obtain good quality images. The measured parameters and mean values for each individual are shown in Table 3, whereas Table 4 presents the comparisons with control values.

**Figure 1:**
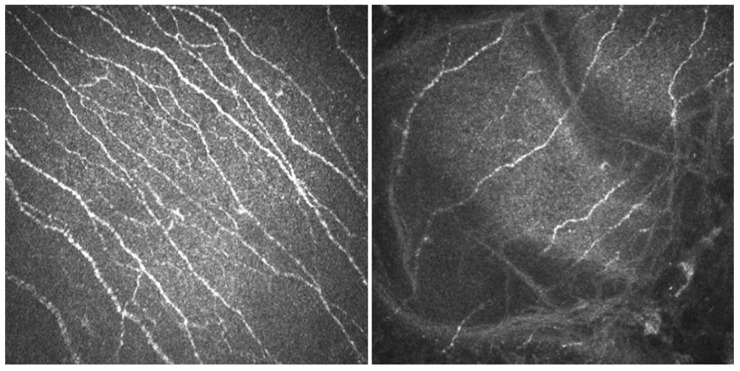
In vivo confocal microscopy images of the central cornea in a healthy control and in a mercury-intoxicated patient. For both corneas, the images were taken at a depth of 50–80 μM. (**Left**) In the healthy control subject, the following measurements were made: nerve density, 15/mm^2^; tortuosity, 2; mean nerve length, 27.94 mm/mm^2^; nerve branch density, 7/mm^2^; dendritic cell density, 4/mm^2^; and reflectivity, 109.1 gray units. (**Right**) In the mercury-intoxicated subject, the following measurements were made: nerve density, 8/mm^2^; tortuosity, 3; mean nerve length, 13.77 mm/mm^2^; nerve branch density, 3/mm^2^; dendritic cell density, 0.5/mm^2^; and reflectivity, 76.29 gray units.

Mercury-intoxicated patients had significantly lower nerve density and nerve branch density than did the controls. Density of dendritic cells in corneal stroma was also decreased in the patients compared to the control subjects. Neuromas were absent in all patients. Nerve length, nerve tortuosity, and reflectivity were not significantly different from controls. Representative images of a patient and a healthy control subject from our files are shown in Figure 1. 

### 3.4. Analysis of Tear Cytokine Concentration 

The percentage of detection and concentration for each tear cytokine is shown in Table 5. Eotaxin, IL-10, IL-4, and MIP-1α were not statistically analyzed any further due to the very low percentage of detection (<30%). For the remaining cytokines, there were no differences in the percentage of detection between the 22 patients and the 22 healthy controls. 

**Figure 2:**
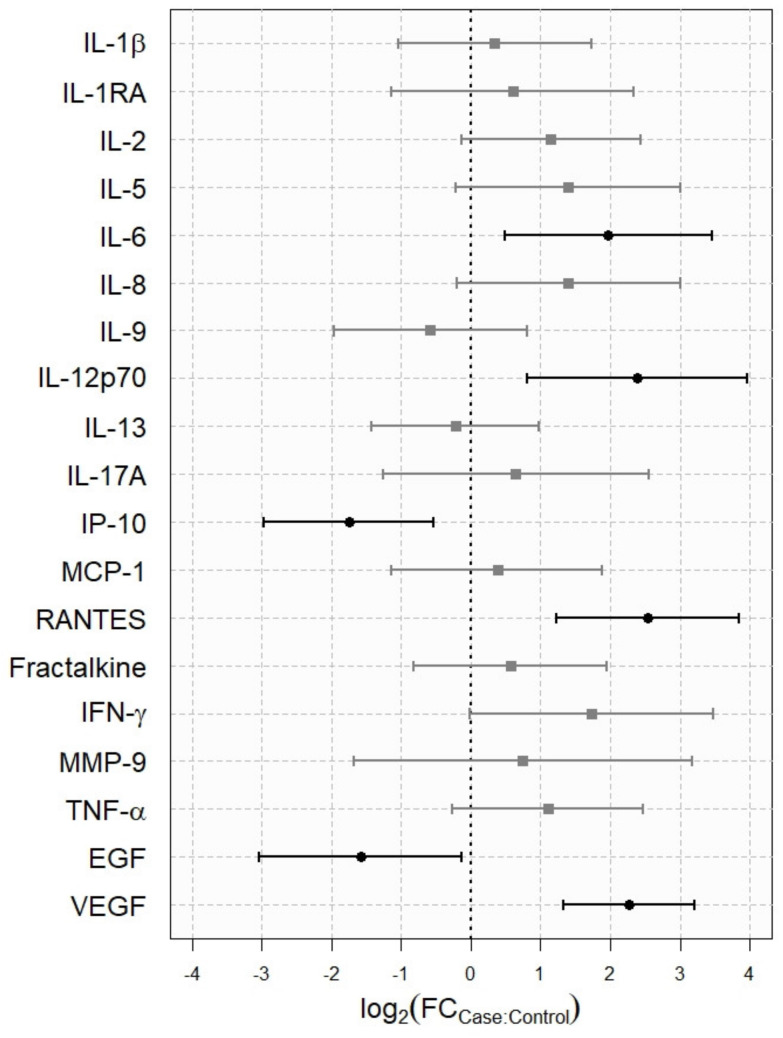
Fold change of tear cytokine levels in mercury-poisoned patients versus healthy controls. Tear cytokines are shown on the *Y*-axis. The fold change (FC) with the 95% confidence interval (CI, horizontal lines) for each cytokine is shown on the *X*-axis. The case: control FC was defined as the relative expression of the cytokine concentration in the patient group divided by the control group. Data on the *X*-axis are presented in a base 2 logarithmic (log2) scale. The vertical dashed line represents no change. The FCs were significant if the 95% CI did not cross the vertical dashed line. The farther the distance to the vertical dashed line, the greater the statistical significance. Positive values mean over-expression and negative values mean under-expression.

For some cytokines, i.e., IL-12p70, IL-6, RANTES, and VEGF, the tear concentrations were significantly higher in the patients than in the control subjects (Table 5, Figure 2). However, for other cytokines, i.e., EGF and IP-10, the tear concentrations were significantly lower in the patients compared to the control subjects.

To further explore correlation patterns among tear cytokine levels in the patient samples, principal component analysis (PCA) and hierarchical agglomerative cluster analysis were performed. To accommodate much of the variance in the primary dataset, PCA was used to build a few independent principal components (PC) based on interrelated levels of the 23 cytokines. In this case, two components explained 81.6% of the sample variability, suggesting that there were two principal components (PC1 and PC2) associated with the tear cytokine levels. PC1 showed high loadings on the levels of RANTES, TNF-α IFN-γ, IL-12p70, IL-5, IL-2, IL-1β, IL-17A, IL-6, VEGF, IL-13, fractalkine, and IL-9. However, PC2 was more correlated with IP-10, IL-8, IL-1RA, EGF, MMP-9, MCP-1, eotaxin, and IL-10 levels.

Based on PC1 and PC2, we then used hierarchical agglomerative clustering analysis to classify the patients into groups. From the resulting dendrogram (grouping tree), two optimal clusters were established. Cluster 1 consisted of 14 patients, (patients no. 2, 6, 7, 9–12, 15–21), and Cluster 2 consisted of 8 patients (no. 1, 3, 4, 5, 8, 13, 14, and 22). The tear cytokine concentrations in Clusters 1 and 2 are shown in Table 6. All concentrations, except those of EGF, IL-1RA, IP-10, and MMP-9, were significantly higher in Cluster 2 compared to Cluster 1. The most increased cytokine concentration in Cluster 2 was IFN-γ which was 43.4 times higher than in Cluster 1 (Table 6).

The comparison of clinical parameter values between patients in Clusters 1 and 2 (Table 7) revealed that the maximum mercury level in urine was significantly higher in Cluster 2 (*p* = 0.0373). Additionally, lysozyme tear levels were significantly lower (*p* = 0.0189) in Cluster 2. There were also significant differences in the density of nerve branching and the density of dendritic cells, both lower in Cluster 2 (*p* = 0.0417 and *p* = 0.0291, respectively). 

## 4. Discussion

In this study, we use a wide variety of techniques to describe for the first time the ocular surface pathology caused by acute/subacute mercury poisoning in 22 male workers who were accidentally exposed to toxic doses of mercury vapor. Briefly, we showed that most patients were highly symptomatic and had increased tear osmolarity, corneal hypoesthesia, altered corneal sub-basal nerve and dendritic cell parameters, and altered tear levels of some inflammation-related cytokines. We concluded that the pathology encountered was consistent with a neurogenic-based DE disease that was more severe in patients with higher urine levels of mercury.

The chief target organ of mercury vapor is the brain, where it causes apoptosis and ischemia of nerve fibers [4]. Damage has also been reported in the peripheral nerves and in the renal, immune, endocrine, and muscle systems [2]. The eye and visual pathways are especially susceptible to neurologically-driven diseases, and the ocular effects of poisoning due to mercury exposure are not unexpected because of the extraordinarily abundant and peculiar innervation of the eye. Four cranial nerves (II, III, IV, and VI) are exclusive to the eye and two others (V and VII) are shared with the rest of facial tissues. Additionally, the retina is one of the most highly specialized nervous tissues in the central nervous system, with unique neurons such as photoreceptors and retinal ganglion cells that send 1.2 million axons through the optic nerve (cranial nerve II), transmitting visual information to the occipital visual cortex. 

The ocular surface also has abundant neural resources, and the cornea is the most highly innervated tissue, not only in the eye, but also in the whole human body. This innervation is sensitive and is delivered by the ophthalmic nerve, which is the first branch of the trigeminal nerve (cranial nerve V) [18]. Because the damage caused by mercury poisoning could target this rich innervation, we evaluated corneal sensitivity and the morphology of the sub-basal corneal nerves by non-contact esthesiometry and IVCM, respectively. Both techniques are minimally invasive, and although not regularly performed in the clinical setting, they can provide invaluable information about some ocular surface diseases. We have accumulated experience with this technique in contact lens-related discomfort [15] and stem cell therapy for corneal pathology [19].

Mercury poisoning, which could be responsible for the neurotoxicity and subsequent damage to the corneal nerves, could also be why the vast majority of the patients had DE-related symptoms, most of which were strongly experienced. Aside from changes in some tear cytokines (as discussed below), there were no signs of alteration in tear production and/or tear quality that could cause epithelial damage to the ocular surface as would be typical in DE. In fact, a disparity between signs visualized with the slit lamp and symptoms is one of the most striking aspects of DE disease and has been reported in many types of DE patients [11,18,20]. This often occurs after corneal refractive surgery (the so-called “pain without stain”) in which there is an unavoidable lesion to the corneal nerves as part of the required laser treatment [21]. In post-refractive surgery patients, and most likely in our mercury-intoxicated patients, neurogenic inflammation due to corneal nerve damage results in the release of the inflammatory mediators [21]. This inflammation could cause the patients to have DE symptoms without manifestation of compromised tear production, and therefore not causing an obvious ocular surface integrity problem.

Tear osmolarity was elevated in 19 of the 22 patients. Although tear hyperosmolarity has been implicated in the pathogenesis of DE [11], there is no obvious explanation as to why the osmolarity was high in these patients, especially since there was no accompanying damage to the integrity of the ocular surface. Consistent with our findings, Yi et al. [22] reported a significant positive correlation between tear osmolarity and ocular symptoms, including cold sensitivity, foreign body sensation, and light sensitivity; however, T-BUT, corneal staining, eyelid hyperemia, and tear secretion volume were not significantly correlated with tear osmolarity. Similarly, Gjerdrum et al. [23] also found tear hyperosmolarity with normal tear production in patients after nerve alteration caused by corneal laser surgery.

Using Belmonte’s gas esthesiometer to measure corneal sensitivity thresholds, we found an increase in the mechanical threshold and in the heat and cold thermal thresholds in mercury-intoxicated patients compared with healthy control subjects. This means that the overall corneal sensitivity was diminished. Benitez del Castillo et al. [20] found similar results for mechanical and thermal sensitivities in Sjögren syndrome-associated DE disease. Likewise, Bourcier et al. [24] reported corneal hypoesthesia with mechanical and thermal stimuli in a more mixed sample of DE patients.

The changes in corneal sensitivity are probably due to the nerve damage that we detected by IVCM. Corneal nerves not only protect the ocular surface through the mechanism of sensation, but they also release trophic factors that regulate wound healing, epithelial integrity, and cell proliferation [16,18]. Thus, nerve damage caused by mercury intoxication could be responsible for an alteration in neuronal stimulation and a delay in the transmission of nerve impulses of the affected fibers. This would explain the decrease in mechanical and thermal sensitivity that we found.

Regarding nerve morphology, corneal nerve density and nerve branch density were significantly lower in the mercury-intoxicated patients, and these changes were associated with higher levels of mercury in the urine of Cluster 2 patients. These results are in agreement with most studies in which there was a significant reduction in the sub-basal nerve density in DE patients compared with controls [25,26]. There are, however, a few studies that show no difference in sub-basal nerve density, but instead, the DE patients had abnormal nerve morphology [27]. Finally, one study of patients with aqueous-deficient DE disease found increased sub-basal nerve density, suggesting the possibility of corneal nerve regeneration in this form of DE [28]. In general, regenerative activity is manifested by nerve branches from endbulbs, and in our patients, the density of branches was diminished. All of these findings support the notion that mercury intoxication adversely affects nerve function and also the capacity for nerve regeneration.

The density of dendritic cells in the corneal stroma of our mercury-intoxicated patients was decreased. Dendritic cells are in contact with the sensory nerve fibers, and play an important role in corneal homeostasis [26,29,30]. Elevated density of dendritic cells is a common finding in inflammatory disorders such as DE disease [26], after refractive surgery [31], in diabetic neuropathy [31], and in infectious keratitis [26]. Consequently, we initially expected to find a higher density of these cells in the cornea of our patients. However, where more centralized nerve damage occurs, such as in patients with fibromyalgia syndrome where the corneal sub-basal nerve plexus is also damaged, corneal dendritic cell density is similarly decreased [29]. In animals, after trigeminal denervation, there is a depletion of dendritic cells, and corneal sensitivity is significantly reduced, delaying corneal recovery during wound healing [30]. So, the decrease in the density of dendritic cells in our mercury-intoxicated patients could be due to the damage we demonstrated in corneal sub-basal nerve plexus. 

Lastly, we found alterations in some tear cytokine levels in the mercury-intoxicated patients, as we expected [2]. Damage caused by mercury intoxication could be responsible for the alterations in nerve stimulation and impulse transmission. It could also cause nerve inflammation, resulting in liberation of several inflammatory cytokines. Indeed, neuro-inflammation is one of the main pathways of methyl mercury-induced central nervous system impairment [2]. Furthermore, in addition to affecting the nervous system, there is accumulating evidence that exposure to mercury alters immunomodulation, although with differences in the mechanism of action depending on the specific form of mercury (inorganic or organic), the species, and even the cell type or tissue [2,32]. Additionally, other cell types apart from nerves, such as ocular epithelial and/or immune cells, could also participate in the ocular surface inflammatory response to mercury exposure. These responses are related to interactions of metals, such as mercury, with electrophilic groups that are not solely restricted to the central nervous system, but are also ubiquitously present in several systems and organs [2,6,7,32].

Other studies have already shown tear molecule alterations in several ocular pathologies [10,12,13,26,33,34,35,36,37,38,39]. Additionally, some studies reported alteration of tear cytokine levels after corneal refractive surgery [40,41,42,43]. While there are several published studies regarding serum and/or tissue cytokine/chemokine levels or gene expression in mercury-intoxicated patients, to our knowledge, this study is the first to address tear cytokine levels in these patients. 

We found that mercury-intoxicated patients had significantly increased tear levels of IL-6, lL-12p70, RANTES, and VEGF, compared to those of the control healthy subjects. Similar findings have been described in DE patients [12,35,44] and in tears from advanced surface ablation refractive surgery patients [40]. The increase in these molecules is in agreement with the increase in serum cytokines in mercury-exposed patients [2], and it reflects an inflammatory response at the ocular surface of these patients.

On the other hand, EGF and IP-10, tear levels were significantly decreased in mercury-intoxicated patients. EGF tear levels usually decrease in DE patients, particularly in the more severe forms [10,36,38,39]. A decrease in tear IP-10 levels has also been described by our group in patients with severe DE associated with ocular graft vs. host disease [39] and by others in primary Sjögren syndrome, in Stevens–Johnson syndrome patients, and in toxic epidermal necrolysis patients [45,46,47]. As IP-10 acts as an inhibitor of neovascularization [48], it has been hypothesized by Yoshikawa et al. [46] that downregulation in IP-10 contributes to the progression of conjunctivalization and neovascularization in Stevens–Johnson syndrome and toxic epidermal necrolysis cases. 

In addition to the measurement of tear cytokine levels in the mercury-intoxicated patients, we performed PC and hierarchical agglomerative cluster analyses to explore correlation patterns among cytokine tear levels and the associations with the clinical findings. Based on the tear cytokine levels, we identified two patient clusters. Patients in Cluster 2 had significantly increased tear levels for 18 out of the 23 cytokines that we assayed, indicating a higher degree of ocular surface inflammation in this group. In agreement with this, the Cluster 2 patients also had significantly decreased tear lysozyme levels, indicating reduced tear production, compared to the patients in Cluster 1. Interestingly, in the same group, the nerve branching density and dendritic cell density were also lower than in Cluster 1. Because the maximum urine mercury levels were significantly higher in patients belonging to Cluster 2, this probably indicates a more intense mercury intoxication in dose and/or exposure time, and/or a higher susceptibility to mercury toxicity [49]. 

In summary, we described a range of unreported ocular surface pathologies produced by mercury poisoning. We hypothesize that the DE-related symptoms experienced by the patients are due to mercury-related damage to the corneal innervation, corneal sensitivity, and tear cytokine disturbances. Thus, the DE-related symptoms and signs associated with mercury poisoning could be described as neurogenic in origin, in contrast to the more classic tear-deficient and/or evaporative-DE subtypes.

## Figures and Tables

**Table 1:** Clinical tests results for mercury-intoxicated male patients.

Patient N°/Age	Onset of Symptoms (Weeks after Exposure)	Osdi (Range 0–100; Normal <12)	Tear Osmolarity (Normal <308 Mosms/L)	Conjunctival Redness (0–4)	T-But (Normal ≥7 s)	Corneal/Conjunctival Staining (Range 0–5)	Schirmer Test (Normal >5 Mm)/Tear Lysozyme Level (Normal ≥1000 μM/mL)	Corneal Sensitivity Thresholds * Mechanical/Heat/Cold
1/45	10	35.00	330	0	2	0/1	5/881	165/+2.16/−1.12
2/29	12	20.80	329	0	10	0/0	25/4934	100/+0.80/−0.80
3/49	1	52.00	323	0	2	1/1	22/1369	200/+0.80/−2.72
4/47	1	84.00	353	0	12	0/0	10/511	160/+2.16/−3.20
5/39	2	14.50	377	0	9	0/0	18/209	90/+1.60/−0.80
6/28	0	14.50	298	0	6	0/0	3/548	50/+1.60/−1.20
7/30	1	50.00	318	0	16	0/0	6/1000	85/+4.00/−4.00
8/37	1	58.30	330	0	9	1/1	4/593	190/+1.60/−2.40
9/50	2	50.00	330	1	7	0/0	11/654	172/+2.80/−3.52
10/44	2	22.90	316	0	16	0/0	25/1415	190/+2.16/−1.44
11/42	2	29.10	338	0	12	0/0	6/629	Not performed
12/52	0	27.00	400	0	14	0/0	15/1186	200/+3.20/−3.52
13/51	3	65.90	323	2	3	1/1	1/391	200/+0.32/−3.20
14/36	4	70.40	332	0	11	0/0	13/153	120/+3.20/−1.20
15/45	1	50.00	356	0	10	0/0	8/316	200/+4.00/−4.00
16/36	2	35.00	330	0	2	0/1	5/881	100/+2.80/−2.20
17/47	1	75.00	338	0	8	2/1	13/1000	142/+2.80/−4.00
18/38	4	12.00	297	0	9	0/0	7/588	200/+3.60/−3.20
19/40	3	27.00	400	0	14	0/0	15/1186	175/+1.20/−2.80
20/42	1	45.00	349	0	12	0/0	6/1849	200/+2.64/−4.00
21/56	2	75.00	342	0	12	1/1	5/760	130/+1.92/−0.56
22/41	1	64.50	288	2	14	1/1	10/316	35/+4.00/−4.00
Mean (SD)	2.54 (2.95)	44.5 (22.04)	336.23 (28.71)		9.55 (4.39)		10.59 (6.97)/970.90 (984.02)	147.81 (53.36)/ +2.35 (+1.10)/ −2.57 (−1.24)
Median [IQR]				0 [0]		0 [0.75]/0 [1]		

SD = standard deviation; IQR = interquartile range; OSDI = ocular surface disease index; T-BUT = tear break-up time.

**Table 2:** Corneal sensitivity thresholds evaluated by non-contact esthesiometry in mercury-intoxicated patients (study group) and in healthy subjects (control group).

	Study Group (*n* = 21)			Control Group (*n* = 22)			
Sensitivity Threshold	Mean (SD)	95% CI		Mean (SD)	95% CI		*p* Value
		Inferior	Superior		Inferior	Superior	
Mechanical (mL/min)	147.81(53.36)	123.52	172.10	69.64 (43.07)	49.49	89.80	0.0001
Thermal hot (°C)	+2.35 (+1.10)	+1.85	+2.85	+1.3 (+0.89)	+0.89	+1.72	0.0018
Thermal cold (°C)	−2.57 (−1.24)	−3.13	−2.00	−1.83 (−1.32)	−2.45	−1.22	0.0470

SD = standard deviation; CI = confidence interval. *p*-values based on comparison of group means by Student’s *t*-test. Bold font denotes statistical significance (*p* < 0.05).

**Table 3:** Corneal morphological data obtained by in vivo confocal microscopy of mercury-poisoned patients.

	Nerve Parameters					
Patient No./Age * (years)	Density (n/mm^2^)	Length (mm/mm^2^)	Tortuosity (0–4)	Density of Branching (n/mm^2^)	Dendritic Cell Density (n/mm^2^)	Reflectivity (Gray Units)
1/45	9.0	10.90	3.0	6.5	3.5	99.70
2/29	8.0	13.77	3.0	3.0	0.5	76.29
3/49	4.0	19.91	3.0	0.5	0.0	92.00
4/47	7.0	16.05	2.0	0.0	14.0	83.97
5/39	4.0	11.39	1.5	0.0	3.0	98.97
6/28	8.0	11.99	2.0	0.5	2.0	88.22
9/50	4.5	9.06	2.0	0.0	12.0	79.30
10/44	3.5	20.51	2.0	1.0	28.5	99.50
11/42	6.0	11.79	1.0	0.0	0.0	93.66
12/52	5.0	19.45	3.0	1.0	4.5	83.42
14/36	2.0	26.15	3.0	0.0	5.0	101.23
17/47	4.0	17.78	2.0	1.0	15.5	118.75
18/38	13.0	16.04	3.0	5.5	23.0	104.56
19/40	8.5	16.81	2.0	6.5	16.5	79.02
21/56	9.5	16.89	2.0	4.5	8.50	97.72
Mean (SD)	6.4 (2.9)	15.90 (4.54)		2 (2.5)	9.1 (8.9)	93.09 (11.56)
Median [IQR]			2.0 [1.0]			

All data are the mean between values acquired by two different researches; *n* = number (15); SD = standard; deviation; IQR = interquartile range. * The number of each patient is the same provided in Table 1.

**Table 4:** Comparison of morphologic cornea parameters obtained by in vivo confocal microscopy in the mercury-intoxicated study group and control values from our group and published literature [14,15,17].

	Study Group Mean (SD) or Median [IQR]	Control Group Mean (SD) or Median [IQR]	*p* Value
Nerve density (n/mm^2^)	6.4 (2.9)	10.5 (3.3) [15]	**0.0006**
Nerve length (mm/mm^2^)	15.90 (4.54)	14.50 (2.90) [17]	0.2151
Density of nerve branching (n/mm^2^)	2.0 (2.5)	52.4 (26.2) [15]	**<0.0001**
Grade of nerve tortuosity (0–4)	2.0 [1.0]	1.9 (0.8) [14]	0.1201
Density of dendritic cells (n/mm^2^)	9.1 (8.8)	57.5 (70.2) [15]	**0.0063**
Reflectivity (Gray units)	93.09 (11.56)	87.16 (13.10) [14]	0.1731

SD = standard deviation; IQR = interquartile range. Student’s *t*-test (parametric) or Mann–Whitney U test (non-parametric). Bold fonts denote statistical significance (*p* < 0.05).

**Table 5:** Percentage of detection and concentration of molecules analyzed in tears of the mercury-intoxicated patients and the healthy controls.

Tear Cytokine	Study Group			Control Group			*p* Value *
	Detection		Concentration pg/mL	Detection		Concentration pg/mL	
	n	% [95%CI]	mean [95%CI]	n	% [95%CI]	mean [95%CI]	
IL-1β	11	50.0 [30.72; 69.28]	18.36 [9.32; 36.16]	10	45.5 [25.07; 67.33]	14.52 [7.07; 29.82]	0.6247
IL-1RA	19	86.4 [64.04; 96.41]	1559.04 [612.89; 3965.78]	12	57.1 [34.44; 77.41]	1031.97[457.15; 2329.54]	0.4923
IL-2	14	63.6 [40.83; 81.97]	38.83 [20.81; 72.44]	6	40 [17.46; 67.11]	17.45 [10.53; 28.93]	0.0761
IL-4	13	59.1 [36.68; 78.52]	nc	6	28.6 [12.19; 52.31]	nc	-
IL-5	10	45.5 [25.07; 67.33]	16.75 [7.10; 39.49]	9	40.9 [21.48; 63.32]	6.36 [2.94; 13.62]	0.0878
IL-6	19	86.4 [64.04; 96.41]	93.53 [56.92; 153.68]	22	100 [81.50; 100]	23.82 [9.4; 60.34]	**0.011**
IL-8/CXCL8	18	81.8 [58.99; 94.01]	62.53 [29.73; 131.53]	11	50 [30.72; 69.28]	23.64 [9.99; 55.88]	0.0856
IL-9	15	68.2 [45.12; 85.27]	32.81 [17.58; 61.24]	9	60 [32.89; 82.54]	49.00 [27.12; 88.54]	0.4011
IL-10	7	31.8 [14.73; 54.88]	nc	2	22.2 [3.95; 59.81]	nc	-
IL-12p70	10	45.5 [25.07; 54.88]	329.58 [204.46; 531.28]	15	68.2 [45.12; 85.27]	63.31 [23.70; 169.13]	**0.0045**
IL-13	19	86.4 [64.04; 96.41]	131.22 [74.39; 231.45]	21	95.5 [75.12; 99.76]	152.63 [80.37; 289.86]	0.7154
IL-17A	10	45.5 [25.07; 67.33]	56.04 [31. 9; 98.48]	4	80 [29.88; 98.95]	35.86 [19.36; 66.43]	0.4916
IP-10/CXCL10	21	95.5 [75.12; 99.76]	6806.46 [3124.56; 14,826.99]	21	100 [80.76; 100]	22,900.94 [16,099.67; 32,575.40]	**0.0063**
MCP-1/CCL2	18	81.8 [58.99; 94.01]	427.52[219.29; 833.51]	10	90.9 [57.12; 99.52]	329.39 [201.37; 538.80]	0.6151
MIP-1αCCL3	4	18.2 [5.99; 41.01]	nc	0	0 [0.00; 34.45]	nc	-
RANTES CCL5	13	59.1 [36.68; 78.52]	354.00 [192.08; 652.42]	17	81 [57.42; 93.71]	61.14 [30.51; 122.50]	**0.0003**
Eotaxin/CCL11	7	31.8 [14.73; 54.88]	n/c	2	18.2 [3.21; 52.25]	nc	-
Fractalkine/ CX3CL1	14	63.6 [40.83; 81.97	1621.12 [838.99; 3132.34]	14	87.5 [60.41; 97.80]	1094.22 [593.23; 2018.30]	0.4125
IFN-γ	13	59.1 [36.68; 78.52]	50.69 [19.26; 133.37]	11	52.4 [30.34; 73.61]	15.25 [7.13; 32.62]	***0.0521***
MMP-9	18	81.8 [58.99; 94.01]	524.80 [205.12; 1342.73]	12	92.3 [62.09; 99.60]	313.56 [92.99; 1057.38]	0.5378
TNF-α	14	63.6 [40.83; 81.97]	24.97 [12.55; 49.68]	11	50.0 [30.72; 69.28]	11.61[5.80; 23.23]	0.1103
EGF	17	77.3 [54.18; 91.31]	445.69 [177.01; 1122.21])	22	100 [81.50; 100]	1333.78 [852.56; 2086.60]	**0.0339**
VEGF	19	86.4 [64.04; 96.41]	4733.30 [3406.12; 6577.61]	10	66.7 [38.69; 87.01]	983.05 [573.75; 1684.33]	**<0.0001**

*n* = number of patients and controls (out of 22 in each group) for whom each molecule was detected; CI = confidence interval; nc = no calculated; IL = Interleukin; IL-1RA = IL-1 receptor antagonist; IP = induced protein; CXCL = chemokine [C-X-C motif] ligand; MCP = monocyte chemoattractant protein; CCL = Chemokine [C-C motif] ligand; MIP = Macrophage inflammatory protein; RANTES = regulated on activation, normal T cell expressed and secreted; CX3CL = chemokine [C-X3-C motif] ligand; MMP = matrix metalloproteinase; TNF = tumor necrosis factor; EGF = epidermal growth factor; VEGF = vascular endothelial growth factor; IFN = interferon. * *p* value corresponding to comparison of concentrations in patient and control groups. Significant *p* values (*p* < 0.05) are denoted in bold.

**Table 6:** Cytokine concentrations in tears of patients in Cluster 1 and Cluster 2.

Tear Molecule	Concentration (pg/mL) Mean (Standard Deviation)/Median [Interquartile Range]		Fold Change (Log2)	Adjusted *p* Value
	Cluster 1	Cluster 2		
IL-1β	12.47 (9.89)/9.94 [11.80]	108.82 (73)/109.45 [73.58]	3.02	**0.0009**
IL-1RA	17918.94 (42897.86)/1120.00 [5331.25]	3075.62 (2899.16)/1455.00 [3310.00]	0.70	0.6152
IL-2	22.39 (18.04)/17.99 [22.57]	197.18 (92.58)/182.00 [43.25]	3.48	**<0.0001**
IL-4	217.01 (191.75)/149.35 [204.63]	1129 (774.36)/1138.50 [1009.25]	2.57	**0.0003**
IL-5	8.95 (8.66)/6.14 [10.52]	175.86 (125.80)/169.00 [141.93]	4.67	**<0.0001**
IL-6	69.05 (51.35)/61.4 [85.19]	305.62 (156.63)/314.50 [124.50]	2.44	**0.0001**
IL-8/CXCL8	289.27 (883.19)/33.35 [72.04]	192.36 (113.63)/158.00 [162.90]	2.21	**0.0144**
IL-9	19.9 (15.13)/16.71 [23.90]	169.93 (107.48)/156.00 [33.68]	3.34	**<0.0001**
IL-10	61.22 (44.61)/53.41 [67.89]	509.64 (415.36)/535.00 [423.75]	2.62	**0.0078**
IL-12p70	195.14 (101.51)/188.51 [172.44]	1163.38 (456.5)/1065.00 [491.75]	2.72	**<0.0001**
IL-13	91.64 (65.76)/88.70 [91.02]	511.62 (256.37)/466.50 [177.75]	2.84	**0.0001**
IL-17A	32.19 (20.14)/29.02 [31.45]	250.71 (118.73)/260.50 [93.50]	3.13	**<0.0001**
IP-10/CXCL10	21,081.29 (33,089.71)/10,660.00 [19,848.25]	13,318.75 (5612.46)/12,800.00 [6342.50]	1.33	0.1529
MCP-1/CCL2	578.88 (1088.12)/237.50 [397.44]	1931.38 (1509.72)/2065.00 [2257.75]	2.50	**0.0083**
RANTES/CCL5	224.68 (214.75)/145.24 [118.81]	1652.62 (613.31)/1625.00 [382.50]	3.37	**<0.0001**
Eotaxin/CCL11	35.72 (54.9)/19.54 [27.23]	218.59 (263.31)/142.70 [212.31]	2.38	**0.0284**
Fractalkine/CX3CL1	1596.81 (3124.23)/643.47 [555.99]	7427.5 (1900.32)/7595.00 [1407.50]	3.38	**0.0001**
IFN-γ	27.2 (34.91)/15.86 [17.22]	650.12 (352.83)/662.50 [316.25]	5.44	**<0.0001**
MMP-9	11,773.76 (40,665.8)/289.00 [1937.85]	1233.25 (902.46)/837.50 [1375.75]	1.42	0.2081
TNF-α	14.18 (11.59)/10.67 [15.54]	154.51 (97.55)/138.50 [48.38]	3.79	**<0.0001**
EGF	2982.49 (8168.04)/145.50 [2205.38]	1728.88 (1192.08)/1895.00 [2116.25]	2.29	0.0981
VEGF	3644.16 (1891.64)/3585.00 [2770.00]	10,180 (3691.33)/10,200.00 [3345.00]	1.60	**0.0002**

IL = Interleukin; IL-1RA = IL-1 receptor antagonist; IP = induced protein; CXCL = chemokine [C-X-C motif] ligand; MCP = monocyte chemoattractant protein = CCL = chemokine [C-C motif] ligand; MIP = macrophage inflammatory protein; RANTES = regulated on activation, normal T cell expressed and secreted; CX3CL = chemokine [C-X3-C motif] ligand; IFN = interferon; MMP = matrix metalloproteinase; TNF = tumor necrosis factor; EGF = epidermal growth factor; VEGF = vascular endothelial growth factor. * *p* value corresponding to comparison of tear cytokine concentration values between Cluster 1 and Cluster 2. Significant *p* values (*p* < 0.05) are denoted in bold.

**Table 7:** Results of Clinical Tests, Esthesiometry, and Corneal Imaging in Patients Classified as Cluster 1 and Cluster 2 Based on Tear Cytokine Levels.

Test	Cluster 1	Cluster 2	*p* Value
OSDI questionnaire (0–100) mean (SD)	38.09 (20.19)	55.58 (21.89)	0.0723
Tear osmolarity (mOsms/L) mean (SD)	338.64 (31.03)	332 (25.56)	0.6137
Conjunctival redness (0–4) median [IQR]	0 [0]	0 [0.5]	0.2295
T-BUT (seconds) mean (SD)	10.57 (3.98)	7.75 (4.77)	0.1517
Ocular surface integrity (0–5) median Corneal staining Conjunctival staining	0 [0] 0 [0]	0.5 [1.0] 1.0 [1.0]	0.1246 0.0656
Tear production mean (SD) Schirmer test (mm/5 min) Lysozyme tear level (μg/mL)	10.71 (7.14) 1209.79 (1143.54)	10.38 (7.15) 552.88 (403.12)	0.8372 **0.0189**
Corneal sensitivity thresholds mean (SD) Mechanical (mL/min) Thermal hot (°C) Thermal cold (°C)	149.54 (51.94) +2.58 (+1.01) −2.71 (−1.31)	145 (59.10) +1.98 (+1.20) −2.33 (−1.17)	0.8555 0.2342 0.4008
Corneal imaging in vivo confocal microscopy Nerve density (n/mm^2^) mean (SD) Nerve length (mm/mm^2^) mean (SD) Nerve branching density (n/mm^2^) mean (SD) Nerve tortuosity (0–4) median [IQR)]Dendritic cell density (n/mm^2^) mean (SD) Reflectivity (Gray units) mean (SD)	7.0 (2.9) 15.41 (3.65) 2.3 (2.4) 2.2 [0.6] 11.10 (9.79) 92.05 (13.46)	5.2 (2.8) 16.88 (6.35) 0.1 (0.3) 2.5 [0.7] 2.88 (2.1) 95.17 (7.20)	0.2786 0.5728 **0.0417** 0.5034 **0.0291** 0.6391
Maximum mercury levels * mean (SD) Blood (μg/L) Urine (μg/g creatinine)	398.57 (273.61) 121.64 (121.65)	359.75 (314.61) 384.75 (396.88)	0.7647 **0.0373**

OSDI = Ocular Surface Disease Index; IQR = Interquartile range. * Mercury normal levels = blood < 10 μg/L and urine < 30 μg/g creatinine. * *p* value corresponding to comparison of concentration values between patient and control groups. Significant *p* values (*p* < 0.05) are denoted in bold.

## Data Availability

Data is contained within the article.
